# The human viral challenge model: accelerating the evaluation of respiratory antivirals, vaccines and novel diagnostics

**DOI:** 10.1186/s12931-018-0784-1

**Published:** 2018-06-22

**Authors:** Rob Lambkin-Williams, Nicolas Noulin, Alex Mann, Andrew Catchpole, Anthony S. Gilbert

**Affiliations:** hVIVO Services Limited, Queen Mary BioEnterprises Innovation Centre, 42 New Road, London, England E1 2AX UK

**Keywords:** Acute respiratory infections; Respiratory medicine; Controlled clinical trial, Pragmatic clinical trials, Research and development, influenza, Flu, Respiratory syncytial virus (RSV), Human rhinovirus (HRV), Human boca virus (HBoV). Therapeutics, Virus-host interactions, Pathogenicity, Immune response, Gene switching, Diagnostic

## Abstract

The Human Viral Challenge (HVC) model has, for many decades, helped in the understanding of respiratory viruses and their role in disease pathogenesis. In a controlled setting using small numbers of volunteers removed from community exposure to other infections, this experimental model enables proof of concept work to be undertaken on novel therapeutics, including vaccines, immunomodulators and antivirals, as well as new diagnostics.

Crucially, unlike conventional phase 1 studies, challenge studies include evaluable efficacy endpoints that then guide decisions on how to optimise subsequent field studies, as recommended by the FDA and thus licensing studies that follow. Such a strategy optimises the benefit of the studies and identifies possible threats early on, minimising the risk to subsequent volunteers but also maximising the benefit of scarce resources available to the research group investing in the research. Inspired by the principles of the 3Rs (Replacement, Reduction and Refinement) now commonly applied in the preclinical phase, HVC studies allow refinement and reduction of the subsequent development phase, accelerating progress towards further statistically powered phase 2b studies. The breadth of data generated from challenge studies allows for exploration of a wide range of variables and endpoints that can then be taken through to pivotal phase 3 studies.

We describe the disease burden for acute respiratory viral infections for which current conventional development strategies have failed to produce therapeutics that meet clinical need. The Authors describe the HVC model’s utility in increasing scientific understanding and in progressing promising therapeutics through development.

The contribution of the model to the elucidation of the virus-host interaction, both regarding viral pathogenicity and the body’s immunological response is discussed, along with its utility to assist in the development of novel diagnostics.

Future applications of the model are also explored.

**Electronic supplementary material:**

The online version of this article (10.1186/s12931-018-0784-1) contains supplementary material, which is available to authorized users.

## Background

This review aims to describe the Human Viral Challenge model; its history, its utility and specifically how it has recently been, and can be used, to potentially accelerate the development of novel therapies for the unmet medical needs of respiratory disease.

## Introduction

Acute respiratory infections (ARIs) manifest as Upper (URI) or Lower (LRI) respiratory tract infections and may move between the two compartments; ARIs represent the most common infectious diseases and are predominantly of viral aetiology. The global burden of ARI is substantial with significant morbidity and mortality occurring in children, the elderly and immunocompromised [1].

In the UK alone during the period 2014-2015, respiratory disease caused an estimated 15,800 excess winter deaths [2]. In the USA, influenza and respiratory syncytial virus (RSV) cause substantial mortality especially among people aged 65 and older [3].

However, although deaths in the industrialised world are widely reported, developing countries feel the burden particularly; out of an estimated 1.9 million child deaths from ARIs in 2000, 70% of those deaths occurred in Africa and south-east Asia [4]. The Millennium Summit at the United Nations in 2000 led to the setting up of the Millennium Development Goals.

A study reported the progress made in meeting those goals in 40 developing countries; it concluded that the prevalence of ARI was 13%, health expenditure and per capita gross domestic product is directly associated with the prevalence of the disease [5].

Viral heterogeneity associated with ARIs is well established [6]. In the past, human rhinovirus (HRV) has been identified as the virus most frequently associated with respiratory illness with 30-50% of infections annually on average, and up to 80% of upper respiratory infections during the autumn outbreaks [7]. After HRVs, coronaviruses (CoV), influenza, respiratory syncytial virus (RSV) and parainfluenza viruses (PIV) are the next most frequent.

More recently an evaluation of illness in 6,266 children under ten years of age in Australia, South East Asia and Latin America emphasised both the viral heterogeneity and the impact of ARI. Of the 2,421 children who experienced 3,717 individual influenza-like Illness (ILI) episodes, rhinovirus/enterovirus was most prevalent (41.5%). Influenza followed this (15.8%), adenovirus (ADV) (9.8%), PIV and RSV (both 9.7%), CoV (5.6%), human metapneumovirus (HMPV) (5.5%) and human bocavirus (HBoV) (2.0%). The percentage of children missing school or childcare was between 21.4% for HBoV and 52.1% for influenza [8].

We have compared the data from the two reports one from 2003 [7] and the other in 2017 [8] and found that the reports, despite being separated by 14 years, were similar, with the single exception of HBoV, discovered in 2005 (Table 1), which we discuss later.

Feng *et al*. [9] described in detail the distribution of ARIs causing hospitalisation by age group: they observed that RSV was predominantly observed in the young and elderly, and influenza although significant in the young was noticeably more predominant in the elderly. Interestingly they observed that co-detection of viruses tended to occur more commonly in the younger age groups, particularly those under the age of five.

**Table 1:** A comparison of two publications separated by over a decade and the incidence of ARI due to viral infection

	Heikkinen et al. 2003 [7]	Taylor et al. 2017 [8]
Rhinovirus	30-50%	41.50%
Coronavirus	10-15%	5.60%
Influenza viruses	5-15%	15.80%
Respiratory syncytial virus	5%	9.70%
Parainfluenza	5%	9.70%
Adenoviruses	<5%	9.80%
Enterovirus	<5%	-
Metapneumovirus	Unknown	5.50%
Unknown	20-30%	0
HBov	NA	2%

NB: The two papers summarised are separated by over a decade therefore different diagnostic methodology were used and are described in each paper. Taylor et al. [8] include Enteroviruses with the rhinoviruses. Hbov was discovered in 2005 and therefore not included by Heikkinen et al. [7]

## Rhinovirus (the “common” cold)

HRV infections, often considered trivial can significantly contribute to missed days from work and school, though infections are typically self-limiting [7]. HRV infections throughout the year and in many cases, manifest with symptoms such as nasal congestion, rhinorrhoea, sneezing, sore throat, and cough. HRV is known to be the primary cause of ARI and a severe contributing factor in exacerbations of atopic disease, e.g., asthma as well other conditions such as chronic obstructive pulmonary disease (COPD) [10–13].

HRV infections are associated with significant economic implications as well as being an important contributor to sinusitis, otitis media, bronchitis and primary pneumonia [14–16]. HRV is a considerable cause of morbidity in specific at-risk groups such as infants, the elderly, immunocompromised, and, as already mentioned, chronic respiratory diseases such as asthma, COPD and cystic fibrosis. At present, HRV is considered the number one cause of asthma exacerbations [15–19].

Asthma is a complex disease, characterised by chronic airway inflammation, and a history of respiratory symptoms such as wheeze, shortness of breath, chest tightness and cough. Over time these symptoms can vary in their intensity [20]. Each year over 300 million people worldwide are affected by asthma: approximately 250,000 people die as a result. Many deaths are due to suboptimal long-term medical care and delay in obtaining help during severe exacerbations of the disease [21]. Treatments to prevent worsening of symptoms and other therapies for mild to moderate asthma that avert relapse, i.e., the symptoms worsen again when the treatment stops, are significant unmet medical needs.

The human challenge model has been used to investigate the viral pathogenicity [22–26] and recent publications on the asthma challenge model have focused on how the asthmatic host responds to HRV infection. Work is ongoing as to susceptibility to viral induced asthma worsening [27, 28] innate immune dysregulation [29] and induction of innate, and type 2 responses in nasal and bronchial epithelial secretions [30].

The pathogenesis of rhinoviral infection, along with other ARIs, in exacerbations of airway disease, has been investigated extensively. Impaired host responses to virus infection, a better understanding of the mechanisms of abnormal immune responses and the potential to develop novel therapeutic targets for virus-induced exacerbations have all used the HVC model [12, 31–34].

Despite previous research work on multiple small molecule antivirals, such as pleconaril which have been tested using both the experimental challenge model and field studies [35–37], there is currently no licensed treatment for HRV infections Other compounds have been tested against HRV, such as Vapendavir (BTA798) which prevented the release of viral RNA into the target cell and demonstrated a reduction in peak viral load in the HVC model [38]. A subsequent study in asthmatics was completed and although not published the compound did have a limited effect [39].

Pirodavir an intranasal capsid-binding molecule reached phase 3 clinical trials for HRV prevention and treatment in the 1990s. Although the compound decreased viral replication and shedding, it failed to show a significant reduction in the duration or severity of symptoms [40, 41].

A Protease inhibitor, rupintrivir thats prevents cleavage of viral proteins required for replication was tested in an HRV challenge trial. Rupintrivir was well tolerated and reduced viral loads and respiratory symptoms [36]. However, in studies of natural infection, it did not significantly affect viral loads or symptom severity [42].

Treatments such as zinc-containing products are now widely discredited as demonstrated by the withdrawal of a Cochrane report and JAMA editorial [43–45].

Current treatment of HRV infections primarily consists of over-the-counter (OTC) medicines to manage symptoms. There is also no licensed vaccine, and while there has been some progress on developing multivalent vaccines [46], development in this area is hampered by the sheer number of serotypes that need to be covered (at present over 160). Despite HRV being associated with up to 50% of adult asthma exacerbations and up to 80% of childhood exacerbations, there are no HRV-specific asthma therapies [34].

As we better understand the interaction between the virus and the host, new therapies such as the monoclonal antibodies (anti-IgE [omalizumab] and anti-IL-5 [mepolizumab]) along with small molecules carefully targeting specific immune signalling pathways, HRV-specific prophylactic treatment may become practical [47–50].

In order to prevent exacerbations, the design of new therapeutics could potentially improve efficacy by both directly acting to inhibit viral replication and alleviate the symptoms of asthma and COPD [51].

## Influenza

Influenza virus is a well-known human pathogen and can cause severe morbidity and mortality, particularly in older patients, those with co-morbidities and in the immunocompromised. In 2009, the first pandemic virus of the 21^st^ century hospitalised 195,000 to 403,000 in the US alone resulting in 8,870 to 18,300 deaths by mid-2010 [52]. A World Health Organization (WHO) global pooled analysis of 70,000 laboratory-confirmed hospitalised H1N1 pandemic patients from 19 countries revealed that of the 9,700 patients admitted to intensive care units, 2,500 died, and that morbid obesity might be a risk factor for hospitalisation and/or death [52]. Obesity was confirmed as a factor associated with a higher likelihood of admission to hospital in influenza-infected patients [53].

The 2009 pandemic was considered mild. However, the classic W shaped age distribution curve of infection for a pandemic virus was observed. That is high mortality in the very young and the old, but an additional spike in death amongst the “young and healthy”. The pandemic, as did previous outbreaks, occurred in successive waves, but despite national policies favouring the use of antiviral drugs, few patients received these before admission to hospital, and many were given antibiotics [54]. The lack of real, or perceived, “real world” efficacy of currently available antivirals leads to the overuse of antibiotics and the subsequent problems that may arise [55–57].

The yearly seasonal morbidity and mortality of influenza results in hospitalisation and death mainly among the high-risk groups. Each year epidemics of seasonal influenza are estimated to result in about 3 to 5 million cases of severe illness, and about 290,000 to 650,000 deaths worldwide [58].

In first world / industrialised countries, most deaths associated with influenza occur among people age 65 or older [59]. Clinics and hospitals, in many countries, can be overwhelmed during peak illness periods, and there can be substantial economic cost [60].

The virus itself has been well characterised, and the two surface proteins, the haemagglutinin (HA) and the neuraminidase (NA) are important in both vaccine and antiviral development [61].

The effects of seasonal influenza epidemics in developing countries are not fully known, but research estimates that 99% of deaths in children under five years of age with influenza-related lower respiratory tract infections are found in developing countries [59, 62].

Currently, vaccines and antivirals exist for the prevention and treatment of influenza, but both have limitations in efficacy due to the rapid evolution of the virus as it mutates on a yearly basis and the sudden unexpected emergence of pandemic influenza strains.

The effectiveness of recent annual influenza vaccines (to date mostly based on the HA, and rarely the NA surface glycoproteins) has languished between 37% and 70% over successive influenza seasons. In particular, the failure of the vaccine across the winter season of 2014-2015, where the overall adjusted effectiveness was 23% [95% confidence interval 14, 31] [63] is memorable. In a mismatched year, the mortality rate is increased in the most at-risk populations [64, 65]. The problem of ensuring that the seasonal vaccine is correctly matched to the upcoming circulating strain highlights the need for rapid development of inter-seasonal/universal vaccines and also the need for a way of testing their efficiency rapidly and accurately before the lengthy and expensive mass production is engaged which takes many months [66, 67].

Antiviral drugs exist of which currently the NA inhibitor oseltamivir is most commonly used. This is active against all known NA subtypes of influenza, and one would, therefore, assume against all influenza strains. They may have decreasing effect with the emergence of resistant influenza strains in which NA protein changes preventing efficient oseltamivir binding and thus its ability to inhibit the essential activity of the viral NA. For example, one genetic mutation known as ‘H275Y’ – a substitution of histidine for tyrosine at NA position 275 - confers an evolutionary advantage to the virus including the 2009 H1N1 influenza [68]. During the 2013-2014 influenza season, 59 (1.2%) of 1,811 influenza A(H1N1) pdm09 virus isolates in 20 of 50 US states had the H275Y oseltamivir resistance substitution. No isolates were resistant to zanamivir [69]. Although animal studies have demonstrated limited transmission of mutant viruses [70, 71], it is thought that the rise of oseltamivir resistance may be due to community transmission [72, 73] rather than the H275Y mutation becoming fixed in the viral genome.

## Respiratory syncytial virus (RSV)

Asystematic systematic review and meta-analysis of published data from 2000 onwards concluded that most RSV-associated child deaths occur particularly in preterm infants and in infants up to 1-year of age [62, 74]. An effective maternal RSV vaccine or monoclonal antibody could have a substantial effect on disease burden in this age group [75].

The RSV-specific monoclonal antibody palivizumab is approved for prevention of serious LRI caused by RSV in susceptible infants. Economic benefit in a UK health setting has not been shown due to the high cost and lack of benefit on serious outcomes [76]. A single-centre cohort study of 22 infants showed no difference in treatment outcomes for patients receiving palivizumab when compared to patients only receiving “standard of care” treatment [77]. Despite the lack of evidence for clinical benefit, post-licensure data supports the use of palivizumab for reducing RSV-associated hospitalisations in premature infants under 33 weeks and in children with chronic lung and heart diseases [78]. Importantly, palivizumab resistant mutant virus has rarely been isolated in clinical specimens [79].

The RSV treatment ribavirin is limited due to difficulty with aerosol delivery, cost and potential harm to healthcare workers, despite off-label treatment of immunocompromised patients being reasonably successful. In the immunocompromised, therapy with a concomitant immunoglobulin or palivizumab has had mixed results, probably due to the difficulty of knowing when to initiate treatment [80].

Despite the call for the accelerated development of prevention and treatment strategies for an effective RSV vaccine for children [81], research has stalled for decades since the death in the 1960s of two subjects in a clinical study. These subjects were infected with a community-acquired RSV infection after receiving the US National Institutes for Health (NIH’s) formalin-inactivated, alum-precipitated RSV candidate vaccine.

In contrast to influenza for which vaccines to date have shown themselves to be moderately effective but in need of improvement, RSV vaccines require substantially more research. There is currently no licensed vaccine for RSV; the most advanced candidate vaccine recently failed to show efficacy in a field study [82]. Effective treatments are urgently required.

RSV is, even amongst healthcare professionals, considered a childhood disease and other confounders have obscured the understanding of the consequences of RSV in adults.

RSV is poorly understood as a disease in the elderly [83], and while the morbidity and mortality in children are of importance, it has been clearly shown that RSV has a comparable health burden to influenza in the elderly [84].

As an example, a recent study was conducted on adult (≥18 years) patients admitted to an emergency department with suspected ARI during 2013–2015 (N = 3743). Multiplex PCR was used to diagnose the cause of the respiratory infection. Eighty-seven patients were identified with RSV. A comparator group with influenza (n=312) was utilised. Based on a 20-day all-cause mortality endpoint, adult patients were less likely to be diagnosed with RSV than with flu (2.3 vs 8.3%, respectively), also they were older, often diagnosed with pneumonia, COPD, hypoxemia, and bacterial co-infection. RSV infection in the elderly was significantly associated with a greater risk of death than seasonal influenza, adjusted for potential confounders and comorbidities. [85]

## Co-infections

The clinical significance of viral/bacterial co-infections has long been a controversial topic. While severe bacterial pneumonia following influenza infection has been well described, associations are less clear among infections caused by viruses common in young children; secondary infections due to other viruses are less well understood and has been reviewed by others [86]. Although assessing the overall contribution of bacteria to disease severity is complicated by the presence of many confounding factors in clinical studies, understanding the role of viral/bacterial co-infections in defining the outcome of paediatric ARI may potentially reveal novel treatment and prevention strategies, improving patient outcomes [33, 86–95].

A recent (2017) publication considered the role of bacterial colonisation with Streptococcus pneumoniae, Haemophilus influenzae and Moraxella catarrhalis during symptomatic and asymptomatic viral upper respiratory infection in the nasopharynx of 4 to 7-year-old children during URI and when well. Using a multiplex PCR, virus was detected in about 80% of upper respiratory tract infections (URIs) in children and is also detectable in the nasopharynx of 30% of asymptomatic children. All three bacteria “levels” were higher during acute URI visits compared to asymptomatic surveillance visits by the children. Of note, however, is that even during asymptomatic follow-up visits, if the virus was present, all bacteria were detected at higher levels [96].

It is worth noting that the presence of confounding infections, can mask the importance of the primary aetiology. Taylor *et al*. [8] report the incidence of HBoV following its identification in 2005 from the respiratory tract samples of children, as an important respiratory pathogen in children. However, the role of this virus on its own as a pathogen of significance was initially unclear, co-infection with other viruses or bacteria was common and confounding.

Moesker *et al*. [97] studied whether HBoV alone could cause acute respiratory infections in children. Using Next Generation Sequencing (NGS), they were able to exclude co-infections amongst those admitted to intensive care unit and studied HBoV viral loads. Of the 990 children who tested positive for a respiratory virus by RT-PCR, HBoV and RSV were detected in 178 and 366 of the children respectively. Forty-nine HBoV-positive patients and 72 RSV-positive patients were admitted to the intensive care. Seven HBoV-infected cases with severe ARI had no other co-infection (7/49, 14%). Importantly, these children did not have another detectable virus as determined by highly sensitive NGS. Also, they had much higher HBoV loads than other patients positive for HBoV, i.e., those with a co-infection. Although small, this study provides strong support that HBoV can cause serious ARI in children with no viral and bacterial co-infections.

## The history of the human viral challenge model

Since Sir Edward Jenner performed the first documented HVC study with smallpox on the 14^th^ of May 1796 the usefulness of such studies has been apparent [98]. More than a century later, Sir Christopher Andrews returned from the US in 1931 he had observed the use of chimpanzees in the study of influenza. The funding for similar work in the UK was insufficient, and therefore Sir Christopher enrolled students from St Bartholomew’s Hospital in London. He explained the next best thing would be a “Bart’s” student as “they were cheaper than chimpanzees”. Over 100 students immediately enrolled, but continued their studies and were not isolated in the same way the chimpanzees had been in the USA [99]. Unfortunately the investigators believed that the symptoms observed may not have been due to the challenge virus, but other respiratory infections acquired in the community, thus confounding the studies. A year later the UK’s Medical Research Council (MRC) terminated the work.

After the conclusion of World War II, the withdrawal of the US troops from the UK left the American Red Cross 'Harvard Hospital' Field Unit on Salisbury plain. The hospital became the Common Cold Unit (CCU) led by Dr David Tyrell, from 1946, volunteers were inoculated by instilling small quantities of the virus into their noses [100]. The CCU housed healthy volunteers in relative isolation from other people, thereby reducing the risk of contact with community-acquired sources of infection or from them passing on the virus to members of the public. The unit was eventually closed in 1989; during four decades of research, it attracted 20,000 volunteers. Its research contributed to a better understanding of respiratory viruses, viral lifecycle, possible vaccines [101] as well as the first licensed anti-influenza compound amantadine [102].

The use of healthy volunteers in the HVC model provided, and still offers, a unique opportunity to describe the viral lifecycle. Investigators know with certainty the time of infection, nasal virus shedding can be measured, symptoms recorded prospectively, and participants are selected with low pre-existing immunity to the challenge virus to ensure a statistically significant infection rate with a small number of volunteers. Thus, such studies can maximise the safety and efficacy data obtained while minimising the risk to study volunteers and limited research funding.

Although serum IgG, for influenza virus, was traditionally measured via the HAI assay, as the entry criteria for volunteers into studies, micro neutralisation assays are used for RSV and HRV. Other work does suggest screening for antibodies to the NA influenza surface protein should be considered [103] or T-cell responses to internal proteins [104] should be considered.

After the closure of the CCU experimental infection studies continued in the USA using small motels and hotels replacing the huts on Salisbury Plain. These studies contributed to the significant development of the new NA inhibitors during the 1990s, including the inhaled drug zanamivir and the orally available drug oseltamivir [105–114].

Studies however also continued in the UK, specifically the University of Southampton who performed important work in atopic volunteers, demonstrating they had more severe colds when experimentally challenged with rhinovirus, than non-atopic controls [115].

The experimental A/Texas H1N1 influenza virus that was used successfully during the 1990s was implicated in the development of myocarditis in an experimentally infected subject, although a causal link was never demonstrated [116]. However, this incident halted work in the USA for a substantial period.

Most, if not all, challenge viruses are manufactured according to Good Manufacturing Practice (GMP) standard. Although controlled nasal inoculation differs from naturally occurring infection – in which exposure to variable quantities of the virus may occur at various mucosal sites - the developed HVC model used in challenge studies mimics natural disease as far as possible [25, 117, 118].

We have described the production of a new GMP stock of virus using an HRV-16 isolate from an 18-year-old experimentally infected healthy female volunteer, provided by colleagues from University of Virginia Children’s Hospital, USA. Importantly, the clinical sample was provided with the appropriate medical history and consent of the donor. We manufactured this new HRV-16 stock by minimal passage in a WI-38 cell line, to reduce the risk of mutations during the Good Manufacturing Practice process. Having first subjected the stock to rigorous adventitious agent testing and determining the virus suitability for human use, we conducted an initial “safety and pathogenicity” clinical study in adult volunteers in a dedicated clinical quarantine facility in London [118].

Our group started HVC studies in the UK in 2001, and since then we have conducted multiple studies with over 2,500 volunteers inoculated with influenza, respiratory syncytial virus (RSV) or human rhinovirus (HRV), and provided numerous proofs of concept [119–121].

## The human viral challenge model: shortening the drug development pathway for ARIs

Influenza, RSV and HRV infection have similar symptomatology, but this differs in severity and predominance of upper, lower or systemic symptoms as has been described by the Center for Disease Control [122]. However, it is not easy to diagnose between the different aetiologies of ARIs, and better diagnostics are needed [123].

Symptoms are common to each infection and manifest on a gradient. Generally, but far from always, influenza infection is more likely to result in a patient feeling so unwell as to take to their bed and have a fever, than RSV, an HRV, CoV or other common cold virus infection, during which daily life is usually less impacted.

A variety of animal models exist to research respiratory viruses such as influenza [124–126], RSV [127–137], HRV [22, 138–140]. No single animal offers a platform for all respiratory viruses that infect humans, and different animal models exist for the same virus, which can give different, often conflicting results.

In addition, the principles of the 3Rs (Replacement, Reduction and Refinement) were developed over 50 years ago to provide guidance and ensure humane animal research. Over time they have become national and international legislation/regulations. The policies of organisations that fund or conduct animal research include these principles as part of the condition of funding [141].

The shared symptomatology of respiratory viruses requires a single standard research platform that can be used to evaluate respiratory disease pathogenesis and the efficacy of candidate therapeutics. The use of a dedicated, purpose-built 24 en-suite bedroom isolation facility in which carefully screened volunteers can be safely inoculated with challenge viruses and intensively monitored may help reduce the use of animals while providing a single consistent research platform with standardised evaluable endpoints for respiratory virus research. Also, we have used a standardised diary card across our studies, which allows for comparison of the symptoms that each virus causes and the efficacy of the therapeutic being tested. We have included a copy of the diary card in the Additional file 1.

It is difficult to evaluate the efficacy of a specific antiviral therapeutic “in the field” due to the presence of circulating community co-infections of differing microbial aetiology. The HVC model allows the opportunity to study a virus in isolation. HVC studies and field studies are complementary research stratagems necessary for the development of effective ARI therapeutics.

**Fig. 1:**
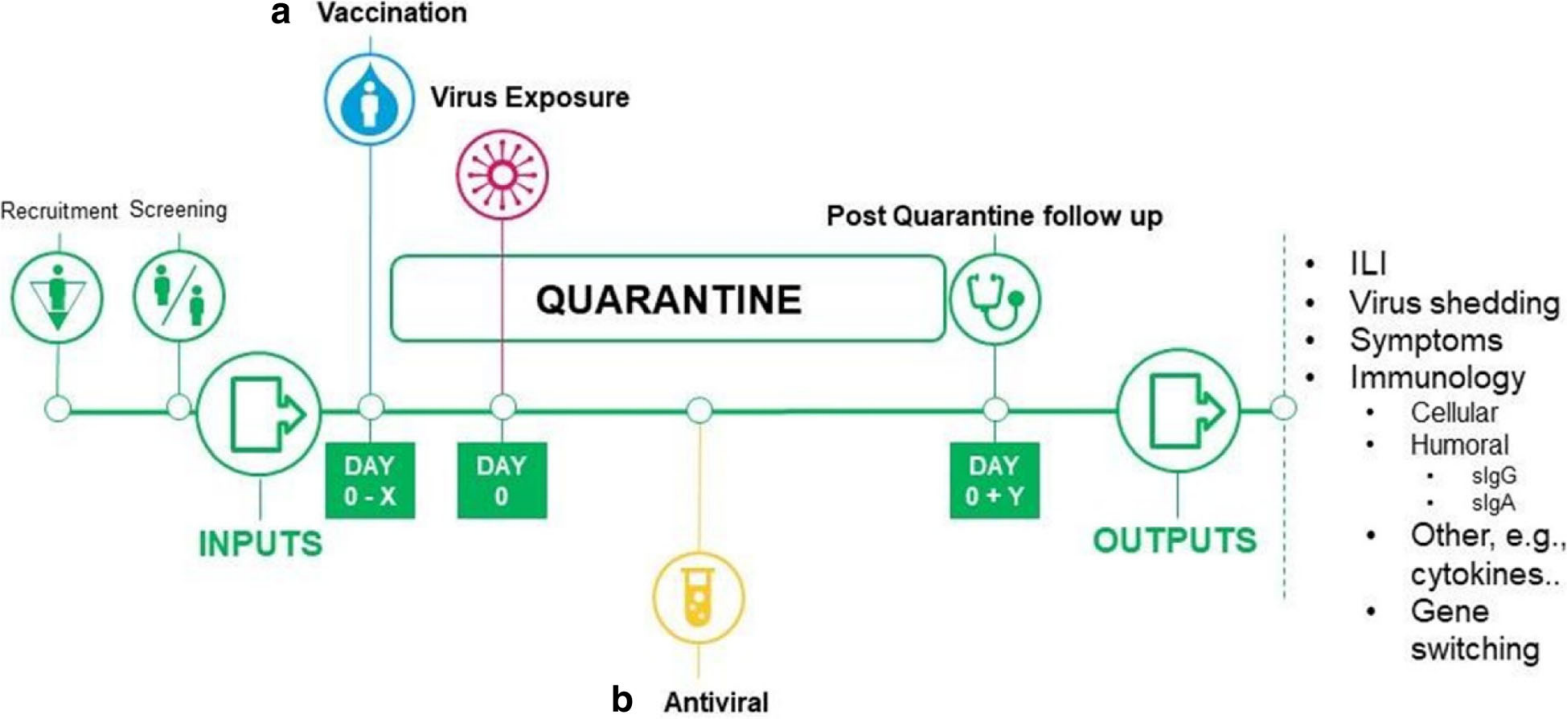
The Human Viral Challenge Model. The study typically consists of inputs, such as the volunteers, their selection criteria, isolation in quarantine and exposure to a GMP virus. There are two treatment options; **a** vaccination/prophylaxis with an antiviral or **b** treatment with an antiviral. Outputs from the study, summarised on the right, such as virus symptoms, virus shedding etc. X is the number of days before virus exposure vaccination may occur. Y is the number of days post virus exposure that a volunteer may be followed for

In contemporary HVC trials, (Fig. 1) healthy volunteers are administered an investigational therapeutic either before (prophylaxis trials) or after (treatment trials) inoculation with the specific challenge strain of the virus. The viruses used in the HVC model are not attenuated and produce symptoms consistent with clinically observed ARI [25, 117, 118]. Each virus is propagated under GMP conditions, with a minimal number of passages from the isolates to the challenge stocks [118, 142]. The few mutations that occur within the virus are rapidly selected out due to a genetic bottleneck, with the consequence that the virus in the human host is considered wild-type [143]. The similarity between virus recovered from the inoculated host and the originator reference virus strain provides assurance that the model disease process is closely aligned with the reference virus strain and is not altered nor attenuated.

There are limited licensed therapeutic options against respiratory viruses, highlighting a significant unmet medical need. A model such as the HVC allows the rapid evaluation of novel therapeutics. The model shortens both preclinical and early clinical development phases by providing a better understanding of the host and pathogen’s initial interaction and has the potential to make the necessary vaccines and medicines more rapidly available than traditional development approaches otherwise might.

**Fig. 2:**
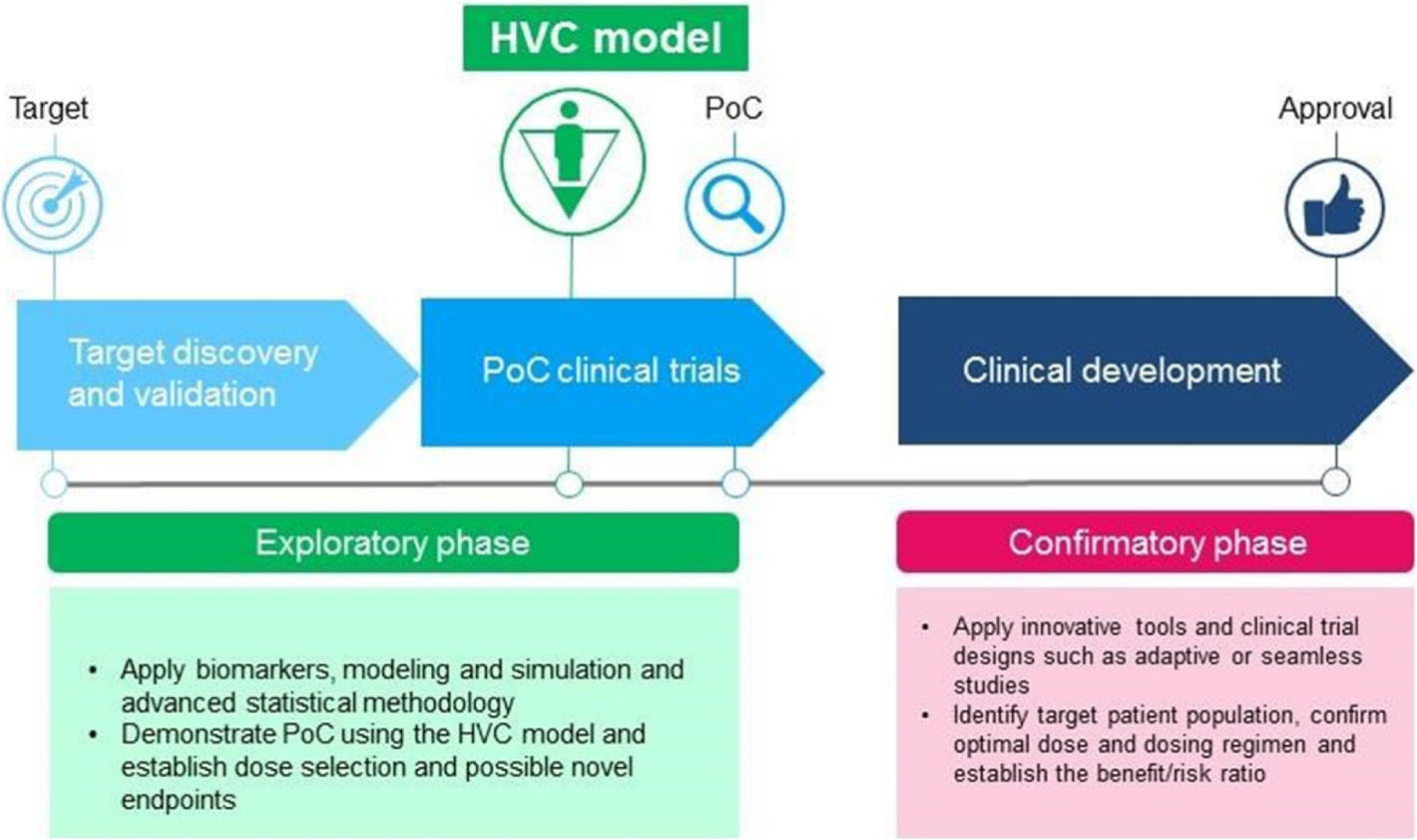
The role of the HVC model in the clinical development pathway. Short duration proof of concept studies, which incorporate the HVC model, typically include small numbers of subjects. The resulting safety and, particularly, efficacy data can more accurately guide decisions on whether to expose a larger number of subjects to promising candidate therapeutics in field studies than conventional phase 1 safety data alone otherwise might

Shortening the traditional development pathway through the early use of a Proof of Concept (PoC) study that incorporates the HVC model (Fig. 2) provides essential evaluable endpoints. Unlike conventional phase 1 studies which rarely include any assessment of efficacy, almost all HVC studies include evaluable efficacy endpoints such as reduction in AUC viral load (mainly recovered from upper respiratory tract samples such as nasal wash or nasopharyngeal swab), volunteer self-reported symptoms, peak symptom score, total symptom score amongst others. Small numbers of subjects – often in the order of 30-45 per treatment group– are typically included in these rapid to execute short duration studies. The resulting safety and pharmacokinetic (PK) and pharmacodynamic (PD) data in controlled conditions, guide decisions on whether or not to progress to field studies, providing a most valuable set of data immediately after, or even as part of, the conventional phase 1 safety study.

The HVC model also opens a different development route alongside traditional phase 1 allowing rapid progress to statistically powered phase 2b studies that will generate the efficacy data needed to support licensing, while still providing suitable safety data. The FDA guidance on developing influenza therapeutics [144] states that challenge trials cannot take the place of efficacy (phase 2) trials. The guidance states; “…Challenge trials can provide useful exposure-response and safety information, as well as an opportunity to demonstrate pharmacological antiviral activity in humans under controlled conditions outside the influenza season. Specifically, data from challenge trials can contribute to dose selection for phase 2b and phase 3 trials, and provide the opportunity to explore the effects of different times of drug initiation relative to virus exposure...”.

Challenge trial refinements are closing the gap between the experimental infection model and the natural infection setting. The HVC study duration of several weeks is shorter than a field-based phase 2 study that waits for a natural outbreak of the virus and the duration of which can be several months/years. These studies save development time when the transition between phases is fully optimised.

Importantly, unlike traditional phase 1b/phase 2 studies, HVC studies are not dependent on a natural outbreak of infection, which can occur at random, and for which the exact time of infection may not be apparent. They provide evaluable endpoints, comparative PD and PK data, along with additional biomarker data on product performance in humans. It must, however, be stated that most often such studies enrol otherwise healthy young adults which imply that the outcome of the infection in the placebo group may be seen as mild to moderate, to some extent. The safety of volunteers has to remain the priority of investigators.

## The HVC model and HRV

The HRV/HVC model can be a potent tool, not just to study HRV infection and disease, but also to investigate the mechanisms of exacerbation in patients with chronic respiratory disease and to conduct efficacy studies for new therapies.

Human challenge studies with HRV have been shown to produce infection in over 90% of serologically susceptible subjects and result in a clinical syndrome that is comparable to that reported with natural colds [145, 146]. Symptoms usually appear within 24 hours and peak at 48-72 hours after inoculation. Virus shedding follows a pattern similar to that of their symptoms. In recent times, several hundred inoculations of adult subjects have been reported and have established this as a safe and effective method in which to study HRV-related disease in both healthy and asthmatic subjects [145].

These studies have provided a knowledge base to further develop the HRV experimental model and provide a controlled and useful tool to develop new therapies for the disease areas associated with HRV infection. New treatments for asthma and COPD are urgently needed, and small animal models of asthma are poorly predictive of efficacy. Most drugs that are effective in these animal models are not found to be effective in later stages of development in humans. Models that more closely follow clinical features of human asthma and COPD are needed [32, 147–151].

## The HVC model and influenza

We have already described current influenza antiviral drugs that can shorten disease and reduce the severity of symptoms if taken early enough after infection, and their prophylactic use can decrease the risk of infection; their utility has been debated however [152].

The two main classes of currently effective anti-influenza drugs are the NA inhibitors, such as zanamivir (Relenza™), oseltamivir (Tamiflu™), peramivir (Rapivab™) [153] and M2 inhibitors, although drug resistance makes this class unusable [154].

The HVC model has recently been used extensively to evaluate new classes of antiviral compounds against influenza, including those such as experimental monoclonal antibodies targeting epitopes within the highly conserved and exposed part of the M2 viral surface protein [155, 156] the conserved stalk of the HA [157] and small molecule antiviral drugs that target the viral polymerase, e.g. favipiravir [158].

## The HVC model and RSV

The HVC model allows for the rapid evaluation of novel therapeutic compounds which may be difficult to evaluate in the field, due to the nature of “at risk” groups, e.g. paediatrics. Specifically, and given the described historical experience with RSV vaccines, it is important that benefit can first be demonstrated in a healthy population.

In the past, unlike influenza and HRV, the HVC model has not been routinely used with RSV. Recently, however, there are several antiviral therapeutics that have reached an advanced stage of development using the model.

We had for some time wished to restart the HVC/RSV studies at the University of London, the two significant challenges that had stalled antiviral development for RSV presented a considerable research need. In association with the DeVincenzo lab at the University of Tenessee and the biotech company Alnylam, we set about designing possibly the first HVC/RSV study.

Alnylam pioneered the use of RNA interference (RNAi) which is a natural mechanism that regulates protein expression and is mediated by small interfering RNAs (siRNA). Working with both groups, we manufactured an RSV Type A virus to GMP standard and titrated it in 35 human volunteers who we divided into five groups, each which was intranasally inoculated with increasing titre (3.0-5.4 log plaque-forming units/person) of the challenge virus. Intranasally. Overall, in this new model, 77% of volunteers consistently shed virus. Infection rate, viral loads, disease severity, and safety were similar between cohorts and were unrelated to the quantity of RSV received.

Symptoms began soon after initial viral detection, peaked in severity near when viral load peaked and subsided as viral loads slowly declined. We concluded that regardless of the titre administered once infections were established the viral load drove illness. We saw no adverse events linked to the virus [25]. Using this new model we conducted an HVC clinical study and demonstrated for the first time that an RNAi had significant antiviral activity against human RSV infection - this established the first-ever proof of concept for an RNAi therapeutic in humans adults [159].

An editorial in the American Journal of Respiratory and Critical Care Medicine, described the utility of the HVC/RSV model saying; “*This model permits the relatively quick and efficient study of new therapeutics in humans and assists in making critical decisions whether to advance a product into costly human trials in populations at highest risk for disease; children, elderly or immunocompromised patients. This constitutes a major and welcome advance in the field of RSV.*” [81]

It is notable that two compounds that have distinct modes of action have recently been evaluated using the HVC model.

First-in-class nucleoside analogue ALS-008176, the efficacy of which was first demonstrated in the HVC model, is currently under evaluation in hospitalised infants [160, 161]. The HVC trial was of randomised, double-blind design, and studied healthy adults inoculated with RSV Memphis 37B [25]. A total of 62 participants received ALS-008176 or placebo for five days after confirmation of RSV infection by PCR (tested twice daily post inoculation). The primary endpoint was the area under the curve (AUC) for viral load post infection. More rapid RSV clearance and a greater reduction in viral load, with accompanying improvements in the severity of clinical disease, were demonstrated in the groups treated with ALS-008176 when compared to the placebo group [160]. Intensive sampling allowed for any potential mutations associated with resistance to be rapidly identified. No such resistant mutations were observed [160].

An RSV-entry inhibitor, GS-5806, a second molecule, first-in-[its]-class was also evaluated. Among the 54 subjects that received active treatment, lower viral load, lower total mucus weight and a lower AUC symptom score were highly significant when compared to placebo [119]. Based on these challenge study data, this therapeutic is now also progressing into potentially pivotal field studies [162].

An essential element of design in both studies was the timing of the first administration of therapeutic post-experimental virus inoculation; the timing was dependent on the detection of virus in nasal wash samples post inoculation of challenge virus by a rapid PCR assay [163], rather than at an arbitrary time point. Subsequently the therapeutic was administered every 12 hours. Careful dose timing, at a clinically relevant point of detection, contributed to the positive outcomes of both studies. It is also believed that by using this “triggered dosing” model, it better mimicked what would happen in a clinical setting as symptoms are known to appear soon after the onset of virus shedding.

## The human viral challenge model for the development of vaccines against respiratory viruses

The HVC model is not limited to novel antiviral compounds but is also important for the evaluation of novel vaccines. Influenza vaccine performance in recent years raises questions about the most appropriate correlates of protection.

Unlike field studies, HVC studies are useful tools for assessing the correlates of protection, vital for vaccine development [103, 104, 164]. Specifically, the importance of the humoral and cellular responses has been highlighted along with the pre-existing T-cell immunity for other respiratory viruses [104].

A recent publication describes the use of the HVC model to demonstrate the efficacy of a novel intranasal proteosome-adjuvanted trivalent inactivated influenza vaccine (P-TIV). In two separate studies, selected subjects who were naïve to A/Panama/2007/1999 (H3N2) virus, were dosed via nasal spray with one of three regimens of P-TIV or placebo. Together, the studies evaluated one or two doses, 15 μg or 30 μg, either once only or twice 14 days apart (1 x 30 μg, 2 x 30 μg, 2 x 15 μg) and subjects were challenged with A/Panama/2007/1999 (H3N2) virus. Immune responses to the vaccine antigens were measured by haemagglutination inhibition (HAI) assay and nasal wash secretory IgA (sIgA) antibodies. Vaccine efficacy was observed ranging from 58% to 82%, comparable to traditional vaccines. The studies also demonstrate that protection against illness associated with evidence of influenza infection significantly correlated with pre-challenge HAI (serum IgG) titres (*p* = 0.0003) and mucosal IgA (*p*≤0.0001) individually, and HAI (*p* = 0.028) and sIgA (*p* = 0.0014) together. HAI and sIgA levels were inversely related to rates of illness. These studies demonstrated the efficacy of this novel intranasal vaccine and answered some important questions concerning true correlates of protection against influenza infection which will help drive future vaccine design. As well as achieving its primary aims, it revealed valuable insights into the correlates of protection and will, we hope, aid future vaccine design [164].

An inter-seasonal or universal influenza vaccine is desperately needed; it will save many lives, whether in those unexpected years when the recommended composition is not matched, or when a pandemic occurs, as it did in 2009. The significance of the 1918 pandemic [165, 166] makes it very clear; up to 100 million people died. A universal vaccine is one that can be prepared for the unexpected, a virus that occurs due to the reassortment of viral genes from different host species.

The HVC model is possibly the only way to initially test such a universal vaccine.

A universal candidate could generate an immune response against the highly conserved virus ion channel protein M2, [167–170], although no vaccine has been shown to be effective in this regard; monoclonal antibodies alone have, the HVC model showed their efficacy [156]. Alternatively, a vaccine may target the conserved stalk of the HA protein [104, 171], or elicit a T-cell response to the internal proteins [172–175]. All are possibilities that have been and can be explored more efficiently using the HVC model.

## Other viruses and future development of the HVC model and novel diagnostics

Although HVC studies provide PoC, researchers, as we have shown, have employed regulatory design standards typical of later phase efficacy studies.

With the development of molecular technology, it is now possible to refine the statistical analysis by stratifying the subjects based on their immune profile. For instance, it is now possible to assess whether a subject is carrying other known respiratory pathogens (bacteria, viruses etc.) and if there is a possible impact on the set of results from the volunteer. Subjects often consent for further analysis of their samples, which allows a valuable biobank of samples to be built for further testing. Moving forward, such samples will allow the use of the HVC model to understand further what happens when a virus infects a person.

It is worth noting that the HVC model is not limited to PoC work on potential therapeutic agents; it is also extensively being used for research purposes, upon which improved treatments for respiratory viruses can be built. In recent years it has been used to demonstrate “gene switching signatures” that could form part of a diagnostic that would reveal infected individuals before they become symptomatic, in the early stages of infection; this could be vitally important in the event of a pandemic [176, 177].

Also, the HVC model has been used to allow a comparison of the relative disease dynamics of different respiratory viruses [24] and to provide a better understanding of the interaction of the virus and the human host [26, 178, 179].

## Conclusions

The HVC model has increased our understanding of the viral life cycle and disease pathogenesis in a tightly controlled setting using small numbers of volunteers. Each volunteer is isolated from each other, and the wider community, ensuring that the disease under consideration is the only one of interest.

The applicability of the virus used to challenge volunteers in the HVC model to a virus that an individual might become exposed to in the “real world” is significant. Whether challenge trials are feasible is dependent on the availability of adequately safety-tested challenge virus strains that are of know providence.

The HVC model provides certain knowledge of the character of the virus; the exact time point of infection; measurability of nasal virus shedding; prospective recording of symptoms and pre-selection of participants for viral challenge who are sero-suitable. This ensures that a statistically significant rate of infection is achieved with the minimal number of volunteers, thus optimising the risk-benefit ratio that supports the determination of therapeutic efficacy.

Crucial to HVC study design is the timing of administration of the first dose of product under investigation to determine optimal effectiveness, not just in the challenge study itself, but in both later stage clinical studies and final clinical use.

The HVC model is an important tool in drug development, in particular with regard to acute respiratory infections. It can accelerate the development of therapeutics that address multiple unmet medical needs. It helps in the understanding of the relationship between a virus and its human host and offers the potential for the development of early-stage diagnostics. It contributes towards identifying new areas for therapeutic intervention. Possibly, and arguably, more importantly, it can ensure that scarce medical resources are directed towards later stage clinical development in an evidence-based manner, and promising therapeutic opportunities are prioritised.

A careful and targeted study design process is a crucial step towards the successful outcome of a challenge trial, because almost all parameters, can be either controlled or at least known (either pre- or post-hoc). Furthermore, results from such trials can be used to make commercial decisions and can lead to major publications, expanding the collective understanding of the scientific community.

Samples from such experiments are of immense value to researchers for the understanding of host interaction mechanisms and the development and validation of therapeutics. Utilisation of consistently collected historical data from HVC studies informs the accurate design and powering of subsequent studies.

HVC studies have been successful in providing proof of concept for DNA vaccines, T-cell vaccines, intranasal vaccines, monoclonal antibodies and small molecules against a range of important respiratory viruses.

It is also encouraging to see that the HVC model is now expanding into further patient populations such as the elderly, asthmatics and those with other conditions such as chronic obstructive pulmonary disease.

An expanding archive of data from preceding studies is an invaluable asset to assist in the selection of volunteers, decide on appropriate endpoints and refine future field study designs.

This allows for safer, statistically sound and more rapidly delivered research.

## Additional file

Additional file 1: Symptom diary card. (PDF 79 kb)
