# Supplementary material for: The human viral challenge model: accelerating the evaluation of respiratory antivirals, vaccines and novel diagnostics
Source: Respir Res. 2018 Jun 22;19:123. doi: 10.1186/s12931-018-0784-1 (PMC6013893; doi:10.1186/s12931-018-0784-1)
Supplement: Supplementary file 1 — Symptom diary card. (PDF 79 kb) [file 12931_2018_784_MOESM1_ESM.pdf]

Symptom Diary Card

Subject Number						Subject Initials			Date									Time (24 hour clock)					Time of Day (mark with an X)		
						F	M	L	D	D	M	M	M	Y	Y	Y	Y	H	H	:	M	M	Morning	Afternoon	Evening

Level	0	1	2	3
Symptoms<br>Please report the symptoms you are experiencing at the moment<br>(Mark with an ×)	I have NO symptoms	Just noticeable	It's clearly bothersome from time to time, but it doesn't stop me from participating in activities	It's quite bothersome most or all of the time, and it stops me from participating in activities
Runny Nose	☐ 0	☐ 1	☐ 2	☐ 3
Stuffy Nose	☐ 0	☐ 1	☐ 2	☐ 3
Sneezing	☐ 0	☐ 1	☐ 2	☐ 3
Sore Throat	☐ 0	☐ 1	☐ 2	☐ 3
Earache	☐ 0	☐ 1	☐ 2	☐ 3
Malaise (tiredness)	☐ 0	☐ 1	☐ 2	☐ 3
Cough	☐ 0	☐ 1	☐ 2	☐ 3
Shortness of breath	☐ 0	☐ 1	☐ 2	☐ 3
Headache	☐ 0	☐ 1	☐ 2	☐ 3
Muscle and/or joint ache	☐ 0	☐ 1	☐ 2	☐ 3

Subject's initials	Doctor's Initials	Date									Time (24 hour clock)				
		D	D	M	M	M	Y	Y	Y	Y	H	H	:	M	M

hVIVO
